# GADD34 suppresses lipopolysaccharide-induced sepsis and tissue injury through the regulation of macrophage activation

**DOI:** 10.1038/cddis.2016.116

**Published:** 2016-05-12

**Authors:** S Ito, Y Tanaka, R Oshino, S Okado, M Hori, K-I Isobe

**Affiliations:** 1 Department of Immunology, Nagoya University Graduate School of Medicine, 65 Tsurumai-cho, Showa-ku, Nagoya, Aichi 466-8550, Japan; 2 Department of Food Science and Nutrition, Nagoya Women's University, 3-40 Shioji-cho, Mizuho-ku, Nagoya, Aichi 467-8610, Japan

## Abstract

Growth arrest and DNA damage inducible protein 34 (GADD34) is induced by various cellular stresses, such as DNA damage, endoplasmic reticulum stress, and amino-acid deprivation. Although the major roles of GADD34 are regulating ER stress responses and apoptosis, a recent study suggested that GADD34 is linked to innate immune responses. In this report, we investigated the roles of GADD34 in inflammatory responses against bacterial infection. To explore the effects of GADD34 on systemic inflammation *in vivo*, we employed a lipopolysaccharide (LPS)-induced murine sepsis model and assessed the lethality, serum cytokine levels, and tissue injury in the presence or absence of GADD34. We found that GADD34 deficiency increased the lethality and serum cytokine levels in LPS-induced sepsis. Moreover, GADD34 deficiency enhanced tissue destruction, cell death, and pro-inflammatory cytokine expression in LPS-induced acute liver injury. Pro-inflammatory cytokine production after LPS stimulation is regulated by the Toll-like receptor 4 (TLR4)-mediated NF-*κ*B signaling pathway. *In vitro* experiments revealed that GADD34 suppressed pro-inflammatory cytokine production by macrophages through dephosphorylation of IKK*β*. In conclusion, GADD34 attenuates LPS-induced sepsis and acute tissue injury through suppressing macrophage activation. Targeting this anti-inflammatory role of GADD34 may be a promising area for the development of therapeutic agents to regulate inflammatory disorders.

Sepsis is a systemic inflammatory disorder caused by bacterial infection. The symptoms of sepsis are fever, mental confusion, transient hypotension, diminished urine output, or unexplained thrombocytopenia.^1^ High levels of pro-inflammatory cytokines in serum, including tumor necrosis factor-*α* (TNF*α*), interleukin (IL) -6 (IL-6), IL-1, and IL-8, are a crucial feature of sepsis.^1, 2, 3, 4, 5^ Inflammation and multi-organ dysfunction are known to be closely related to sepsis-induced mortality.^1, 2, 3^ Despite robust previous research on the pathophysiology of sepsis, this syndrome remains the chief cause of death in intensive care units.^1, 2, 4^

Recently, the innate immune system's ability to detect the invasion of pathogenic microorganisms has been elucidated. Toll-like receptors (TLRs) have a major role in pathogen recognition and the initiation of inflammatory and immune responses.^6, 7, 8^ In particular, the signaling pathways of TLR4, which recognizes bacterial lipopolysaccharide (LPS), are well established. TLR4 triggers the activation of the myeloid differentiation primary-response protein 88 (MyD88)-dependent and MyD88-independent pathways.^8^ The MyD88-dependent pathway is regulated by MyD88 and TIR-domain-containing adaptor protein (TIRAP), and activates the transcriptional factor nuclear factor-*κ*B (NF-*κ*B), that in turn promotes the expression of pro-inflammatory cytokines.^8, 9, 10^ The activation of NF-*κ*B is inhibited by the inhibitor of NF-*κ*B (I*κ*B) family. LPS initiates the activation of the I*κ*B kinase (IKK) complex, which phosphorylates and ubiquitinates I*κ*B proteins and promotes their subsequent degradation by the proteasome, and the concomitant release and nuclear translocation of the NF-*κ*B complexes.^8, 9, 11, 12^ The activation of the MyD88-independent pathway initiates interferon (IFN)-*β* production through the activation of the transcriptional factor interferon-regulatory factor 3 (IRF3) via the phosphorylation of IKKɛ and TANK-binding kinase 1 (TBK1).^13, 14, 15, 16, 17^

Growth arrest and DNA damage inducible protein 34 (GADD34) was originally isolated based on ultraviolet-inducible transcripts in Chinese hamster ovary cells.^18^ The expression of GADD34 is induced by several cellular stresses, such as DNA damage, endoplasmic reticulum (ER) stress, and amino-acid deprivation.^19, 20, 21, 22^ Recently, it was reported that GADD34 is linked to cytokine production in response to viral infection. TLR3 ligation, induced experimentally through poly(I:C) stimulation, induced GADD34 to promote cytokine production, such as IFN-*β* and IL-6, through eukaryotic initiation factor 2*α* (eIF2*α*) dephosphorylation.^23, 24^ However, the role of GADD34 in innate immune responses against bacterial infection is still unclear. In this report, we describe a novel role for GADD34 in inflammatory responses against bacterial infections and identify the signaling mechanism through which it works.

## Results

### GADD34 protects against LPS-induced sepsis

**Figure 1:**
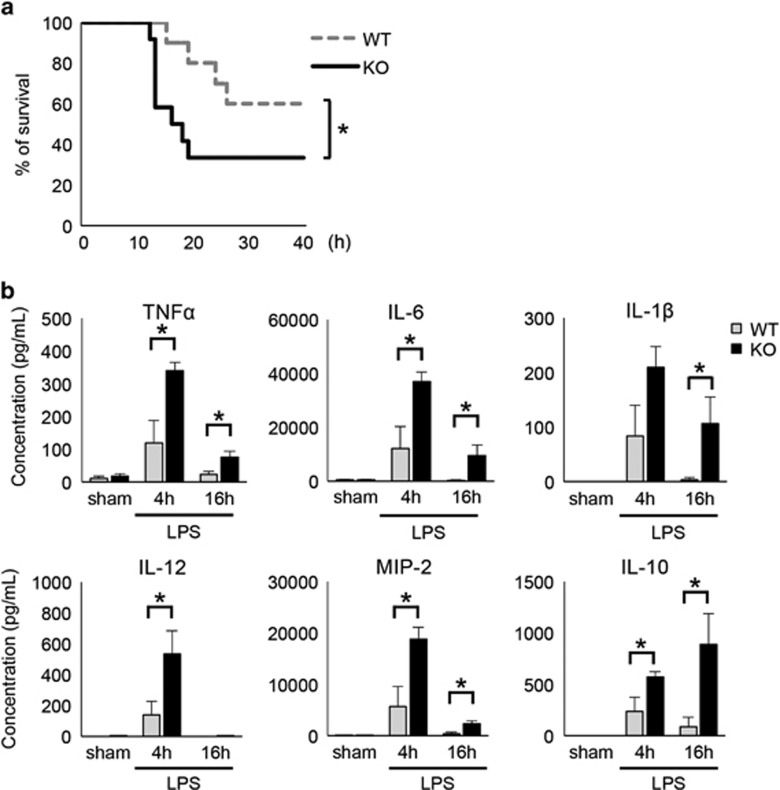
Effect of GADD34 on LPS-induced lethality and cytokine production. (**a**) Survival curves after 30 mg/kg body weight LPS injection (WT: *n*=10, GADD34KO: *n*=12). *P<0.05 (Wilcoxon test). (**b**) Cytokine concentrations in serum at 0 (sham), 4, and 16 h after LPS injection (5 mg/kg body weight). Data represent means±S.E.M. (*n*=4–7/group). *P<0.05

To investigate the effect of GADD34 on LPS-induced sepsis, WT mice and GADD34KO mice were injected intraperitoneally with a high dose (30 mg/kg body weight) of LPS. We found that the survival of GADD34KO mice was significantly lower than that of WT mice, especially soon after LPS challenge (Figure 1a). Next, we treated WT and GADD34KO mice with 5 mg/kg LPS and measured serum cytokine levels. In WT mice, serum concentrations of pro-inflammatory cytokines, including TNF*α*, IL-6, IL-1*β*, IL-12, and macrophage inflammatory protein-2 (MIP-2), which is an ortholog of human IL-8, were markedly increased at 4 h, and decreased at 16 h, after LPS treatment (Figure 1b). GADD34KO mice showed significantly higher production of these cytokines compared with WT mice after LPS injection (Figure 1b). In addition, the anti-inflammatory cytokine IL-10 was also higher in GADD34KO mice than in WT mice after LPS administration (Figure 1b). These results show that GADD34 suppressed LPS-induced sepsis and inflammatory cytokine production.

### GADD34 attenuates acute liver injury induced by LPS

**Figure 2:**
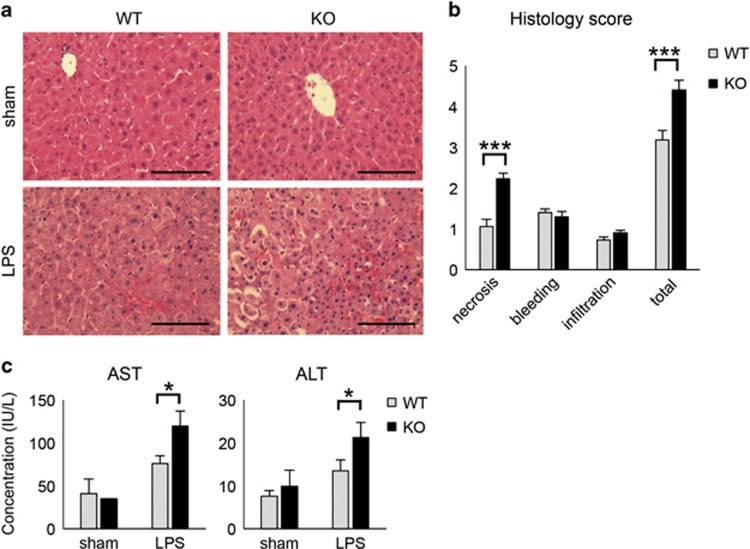
Loss of GADD34 enhances LPS-induced acute liver injury. WT and GADD34KO mice were treated with or without LPS (5 mg/kg body weight). After 16 h of injection, liver samples and serum were harvested. (**a**) Representative images of H&E staining of mouse liver sections. Scale bar: 100 *μ*m. (**b**) Semiquantitative scoring of histopathology. (**c**) Serum AST and ALT levels. Data represent means±S.E.M. (*n*=7). *P<0.05, ***P<0.001

We next employed an LPS-induced acute liver injury in order to assess the effect of GADD34 on tissue injury. Sixteen hours after LPS treatment, the livers of GADD34KO mice exhibited more widespread destruction than those of WT mice (Figure 2a). Histological scoring of H&E-stained liver sections revealed that there were no differences in the amount of infiltrating cells in the liver or the level of bleeding between WT and GADD34KO mice (Figure 2b). However, the livers of GADD34KO mice exhibited far greater necrotic areas than those of WT mice (Figure 2b). Moreover, GADD34KO mice showed significantly higher aspartate aminotransferase (AST) and alanine aminotransferase (ALT) levels in their sera than WT mice (Figure 2c). The expression of CyclinD1, which is related to hepatocyte regeneration after liver injury, was higher in livers from GADD34KO than WT mice after LPS treatment (Supplementary Figure S1A). These results indicate that GADD34 attenuated acute liver injury induced by LPS.

### GADD34 inhibits LPS-induced ER stress responses and hepatic apoptosis

**Figure 3:**
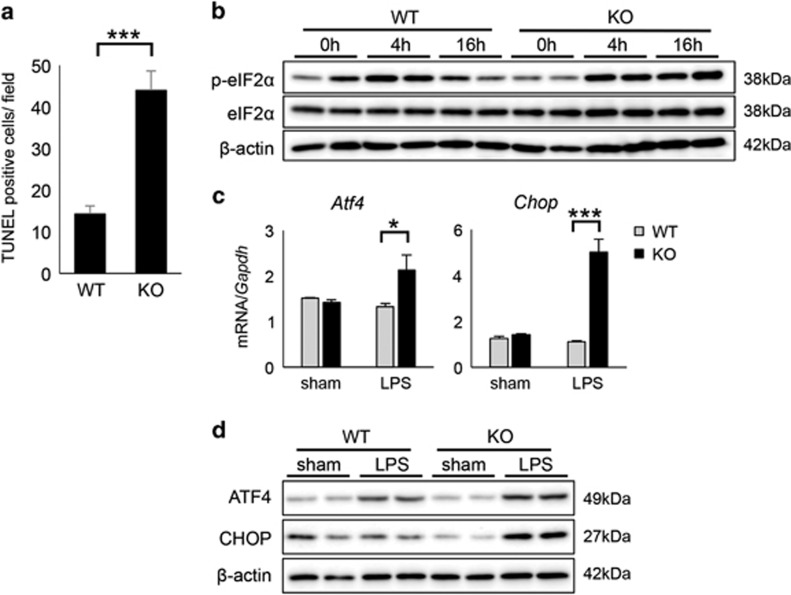
Loss of GADD34 enhances hepatocyte apoptosis after LPS treatment. WT and GADD34KO mice were treated with or without LPS (5 mg/kg body weight). After 16 h of injection, liver samples were harvested. (**a**) The number of TUNEL-positive cells in × 100 magnification field. (**b**) Western blotting analysis of liver samples. The original immunoblots are presented in Supplementary Figure S7. (**c**) Real-time PCR analysis of liver samples. (**d**) Western blotting analysis for ATF4 and CHOP expression of liver from mice treated with LPS for 4 h. The original immunoblots are presented in Supplementary Figure S7. Data represent means±S.E.M. (*n*=4). *P<0.05, ***P<0.001

To test whether GADD34 affects hepatic apoptosis, we evaluated apoptosis by TUNEL staining. We found that GADD34 inhibited apoptosis in liver at 16 h after LPS injection (Figure 3a and Supplementary Figure S1B). GADD34 is known to regulate eIF2*α* phosphorylation, which is the response to ER stress.^25, 26^ We observed that phosphorylation of eIF2*α* in WT liver was upregulated by LPS at 4 h, and it was decreased at 16 h after LPS treatment (Figure 3b). However, the liver of GADD34KO mice exhibited a prolonged upregulation of eIF2*α* phosphorylation, and showed much higher phosphorylation of eIF2*α* than WT liver at 16 h after LPS injection (Figure 3b). We measured the expression levels of protein kinase RNA-like endoplasmic reticulum kinase (PERK), which phosphorylates eIF2*α*, in the liver. Although there was a trend toward increased expression of *Perk* mRNA by LPS treatment, there was no significant difference between WT and GADD34KO livers (Supplementary Figure S1C). We also analyzed the expression of activating transcription factor 4 (ATF4) and C/EBP homologous protein (CHOP), which are the downstream targets of eIF2*α* in the liver. Real-time PCR analysis revealed that the expression of *Atf4* mRNA in GADD34KO livers was significantly higher than that in WT livers at 16 h after LPS treatment (Figure 3c). Similarly, GADD34KO liver showed much higher mRNA expression of *Chop*, which promotes apoptosis, than WT liver after 16 h of LPS exposure (Figure 3c). Western blotting analysis also showed that the upregulation of ATF4 and CHOP protein expression following LPS treatment was higher in GADD34KO liver than in WT liver (Figure 3d). Taken together, these results indicate that GADD34 inhibited hepatic apoptosis through downregulating the eIF2*α*-ATF4-CHOP pathway induced by LPS.

### GADD34 attenuates the production of pro-inflammatory cytokines in liver

**Figure 4:**
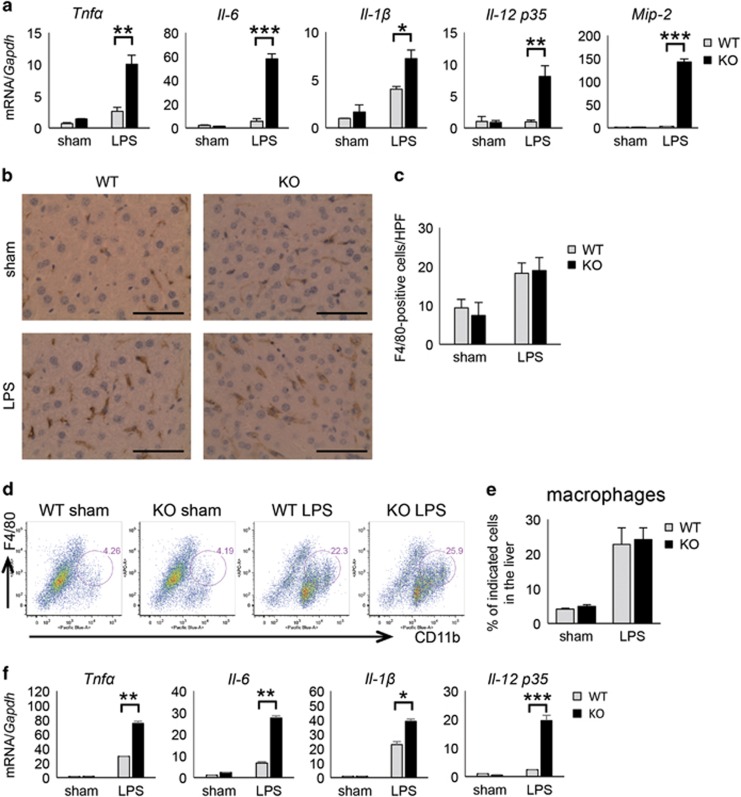
GADD34 regulates cytokine production in the liver tissue. WT and GADD34KO mice were treated with or without LPS (5 mg/kg body weight). Sixteen hours after injection, liver samples were harvested. (**a**) Real-time PCR analysis for inflammatory cytokines from liver. (**b**) Representative images of immunohistochemistry for F4/80^+^ macrophages in the liver. Scale bar: 50 *μ*m. (**c**) Number of F4/80-positive cells in the liver of sham and LPS-treated cells at 16 h. (**d**) Representative flow-cytometric data plot of liver samples. Sixteen hours after LPS injection, liver samples were harvested and analyzed by FACS. Liver samples were stained with eFluor450-conjugated anti-CD11b, and APC-conjugated anti-F4/80 antibodies. (**e**) The numbers of F4/80^+^/CD11b^+^ macrophages in the liver 16 h after LPS injection were determined by flow-cytometric analysis. (**f**) Kupffer cells were isolated from the livers of GADD34KO or WT mice treated with or without LPS (5 mg/kg body weight) for 4 h. 100 ng total RNA of Kupffer cells was used for the synthesis of first-strand cDNA. Cytokine expression levels were determined by real-time PCR analysis. Data represent means±S.E.M. (*n*=4). *P<0.05, **P<0.01, ***P<0.001

Next, we measured the expression of pro-inflammatory cytokines induced by LPS in the liver. Sixteen hours after LPS injection, GADD34KO mice showed much higher expression of *Tnfα*, *Il-6*, *Il-1β*, *Il-12 p35*, and *Mip-2* mRNA than WT mice (Figure 4a). To investigate the function of GADD34 in other organs, we analyzed LPS-induced inflammation in kidney and lung by H&E staining or real-time PCR analysis. As with the liver, kidney from GADD34KO mice presented more severe injury than kidney from WT mice (Supplementary Figure S2A). In addition, although the infiltration into the LPS-treated lung in GADD34KO mice was the same as in WT mice (Supplementary Figure S2B), the mRNA expression of *Tnfα*, *Il-6,* and *Il-1β* in LPS-treated lung was much higher in GADD34KO mice than in WT mice (Supplementary Figure S2C). As cytokines such as TNF*α* and IL-6 are mainly produced by myeloid cells, we next quantified the number of infiltrating F4/80-positive macrophages in LPS-treated liver by immunohistochemistry. Although LPS treatment induced the infiltration of F4/80-positive macrophages into the liver, the absence of GADD34 did not affect their infiltration (Figures 4b and c). FACS analysis supported that there is no difference in LPS-induced infiltration of F4/80^+^/CD11b^+^ macrophages into the liver between WT and GADD34KO mice (Figures 4d and e). Taken together, loss of GADD34 enhanced the production of pro-inflammatory cytokines in the liver, but did not increase myeloid cell infiltration. These results suggest that GADD34 might reduce pro-inflammatory cytokine production by suppressing the activation of myeloid cells. To understand whether GADD34 suppressed inflammatory cytokine production from myeloid cells, we next examined cytokine production by Kupffer cells in WT and GADD34KO mice. Immunofluorescence studies revealed that F4/80-positive Kupffer cells in GADD34KO liver expressed higher TNF*α* and IL-6 than in WT liver (Supplementary Figure S3). Moreover, we found that isolated Kupffer cells from LPS-treated GADD34KO liver had higher mRNA expression of cytokines such as *Tnfα*, *Il-6*, *Il-1β,* and *Il-12 p35* than those from LPS-treated WT liver (Figure 4f). Thus, these results indicate that GADD34 inhibits LPS-induced inflammation through suppressing pro-inflammatory cytokine production by macrophages.

### GADD34 regulates the production of pro-inflammatory cytokines from macrophages

**Figure 5:**
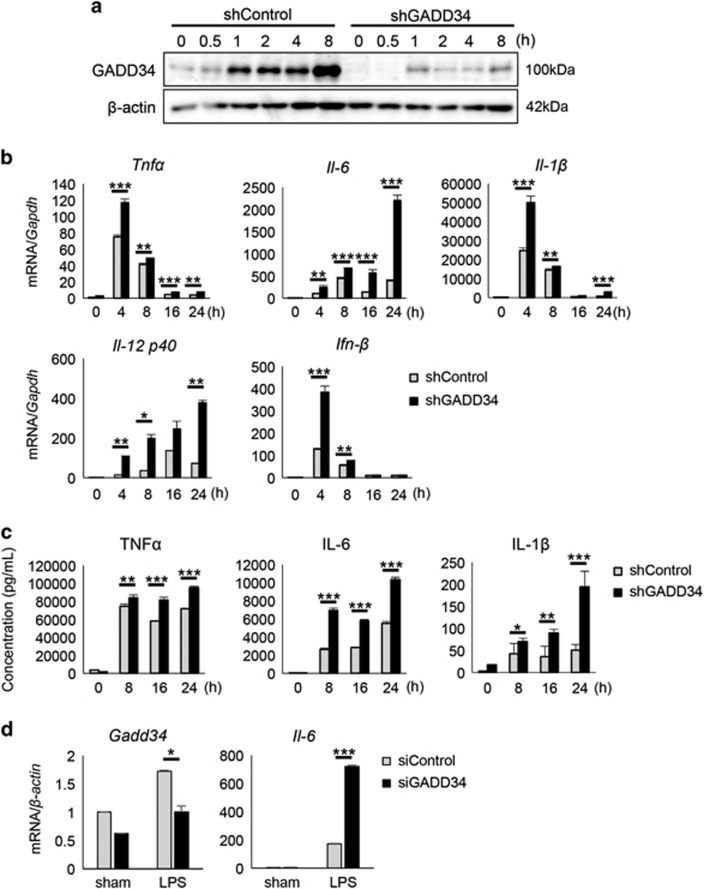
GADD34 deficiency increases cytokine production from RAW264.7. RAW246.7 cells were knocked down for GADD34 expression. GADD34 knocked-down RAW264.7 cells (shGADD34) and control RAW264.7 cells (shControl) were treated with LPS (1 *μ*g/ml). (**a**) Western blotting analysis for GADD34 expression of shControl and shGADD34 treated with LPS. Representative of three separate experiments. The original immunoblots are presented in Supplementary Figure S7. (**b**) Real-time PCR analysis for inflammatory cytokine expression. (**c**) ELISA analysis for inflammatory cytokine expression in supernatants of shControl and shGADD34. (**d**) The expression of GADD34 in THP-1 cells was knocked down by siRNA, and the expression of cytokines after LPS (1 *μ*g/ml) stimulation for 4 h was determined by real-time PCR analysis. Data represent means±S.E.M. (*n*=3). *P<0.05, **P<0.01, ***P<0.001

To clarify the effect of GADD34 on the activation of macrophages, we knocked down the expression of GADD34 by shRNA treatment (shGADD34) in the macrophage cell line RAW264.7, and then stimulated them with LPS. Western blotting analysis revealed that shGADD34 treatment successfully knocked down GADD34 expression (Figure 5a). On the other hand, the expression of GADD34 was markedly increased by LPS in cells treated with non-target control shRNA (shControl) (Figure 5a). Using GADD34-deficient RAW264.7 cells and control RAW264.7 cells, time-dependent changes in the cytokine expression profile of these cells after LPS stimulation were measured by real-time PCR analysis and ELISA. The production of pro-inflammatory cytokines, including *Tnfα*, *Il-6*, *Il-1β*, *Il-12 p40,* and *Ifn-β*, was increased by LPS treatment, and they were much higher in GADD34-deficient cells than in control cells at nearly all time points after LPS stimulation (Figures 5b and c). Similar results were obtained by stimulating with other TLR ligands. After GADD34-deficient and control cells were stimulated with the indicated TLR1–9 ligands (TLR1/2: Pam3CSK4, TLR2: HKLM, TLR3: Poly(I:C)LMW or Poly(I:C), TLR4: LPS-EK, TLR5: ST-FLA, TLR6/2: FSL-1, TLR7: ssRNA40, TLR9: ODN1826), GADD34 expression was analyzed by western blotting, and the production of pro-inflammatory cytokines from these cells was measured by ELISA. The expression of GADD34 was induced by all of the TLR ligands (Supplementary Figure S4A). TNF*α* and IL-6 production was increased by stimulation with the TLR ligands, and GADD34-deficient cells showed higher levels of these cytokines than control cells by almost all of the TLR ligands except TLR3 ligands (Supplementary Figure S4B). In another experiment using murine primary peritoneal macrophages from GADD34KO and WT mice, we obtained similar results for pro-inflammatory cytokine production as in the experiment using RAW264.7 cells (Supplementary Figure S5A). In addition, in order to investigate the effects of GADD34 on inflammation in humans, we examined the ability of the human monocytic cell line THP-1 to produce cytokines in the presence or absence of GADD34. The expression of GADD34 was knocked down using siRNA, and the GADD34 downregulation was confirmed (Figure 5d). Subsequently, GADD34-knockdown and control THP-1 cells were stimulated with LPS, and *Il-6* mRNA expression was assessed by real-time PCR analysis. As with our murine macrophage experiments, GADD34-knockdown THP-1 cells showed significantly higher *Il-6* mRNA expression than control THP-1 cells (Figure 5d). Collectively, these data indicate that GADD34 has a critical role in inhibiting the production of pro-inflammatory cytokines from macrophages and suppressing the inflammation induced by bacterial infection.

### GADD34 inhibits TLR signaling in macrophages through dephosphorylation of IKK*β*

**Figure 6:**
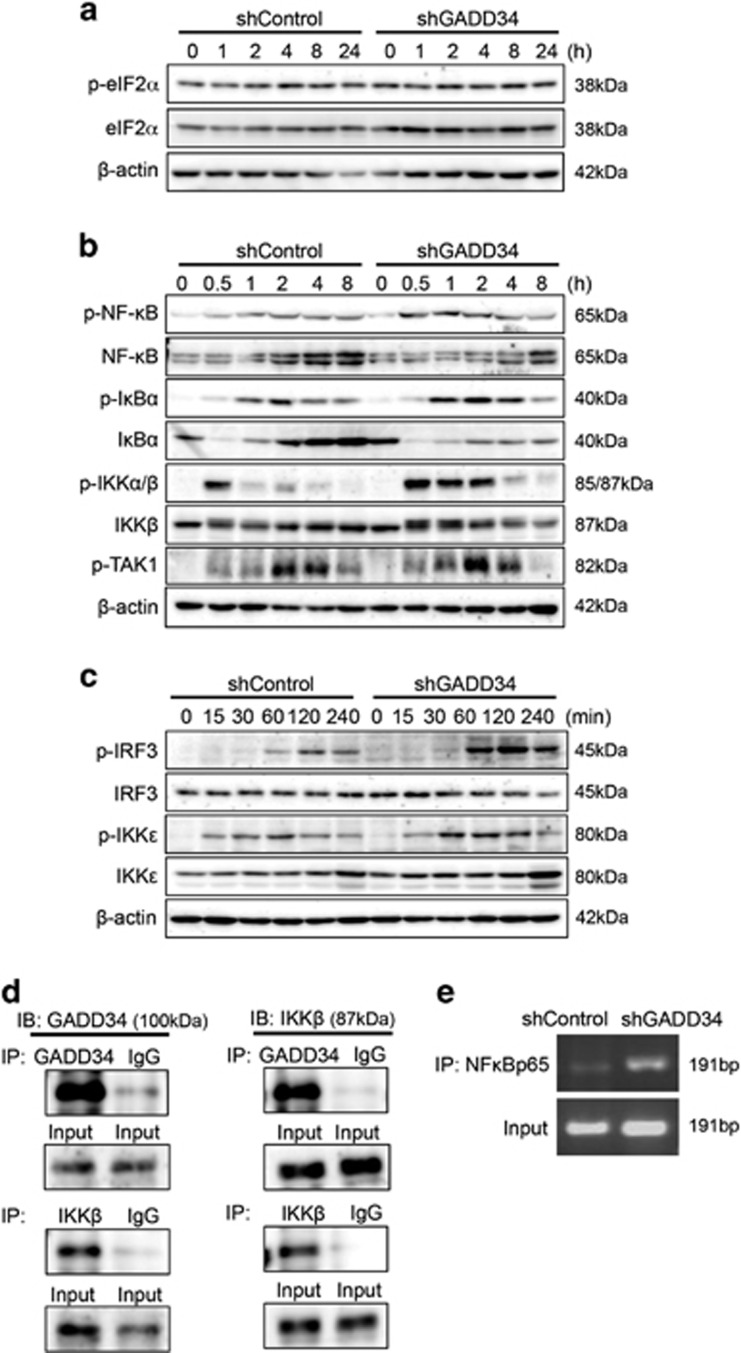
GADD34 deficiency enhances TLR4 signaling in RAW264.7 cells. (**a**–**c**) Western blotting analysis of shControl and shGADD34 treated with LPS (1 *μ*g/ml). (**d**) Immunoprecipitation analysis. Cell lysates of control RAW264.7 cells were subjected to immunoprecipitation using anti-GADD34 or anti-IKK*β* antibody, or anti-rabbit IgG antibody. Immunoprecipitates were analyzed by western blotting with anti-GADD34 and anti-IKK*β* antibody. (**e**) Chromatin immunoprecipitation analysis of NF-*κ*B p65 recruitment to the *κ*B sites of the IL-6 promoter regions performed on shControl and shGADD34 cells treated with LPS (1 *μ*g/ml) for 4 h. These data are representative of three separate experiments. The original data are presented in Supplementary Figure S8–S10

To elucidate the mechanism of how GADD34 inhibits macrophage activation by LPS, we analyzed ER stress responses and TLR4 signaling by western blotting. The level of eIF2*α* phosphorylation was not changed by LPS stimulation in either GADD34-deficient or control cells (Figure 6a). This result indicates that ER stress did not affect the activation of macrophages after LPS stimulation. It is well known that TLR4 signaling activates the transcriptional factor NF-*κ*B p65 and promotes the production of pro-inflammatory cytokines. Therefore, we analyzed the TLR4-mediated NF-*κ*B signaling pathway. LPS stimulation induced higher phosphorylation of NF-*κ*B in GADD34-deficient cells than in control cells (Figure 6b). In addition, the activation of IRF3, a transcription factor of IFN-*β*, and its upstream IKKɛ were both higher in GADD34-deficient cells than in control cells after LPS stimulation (Figure 6c). Furthermore, I*κ*B*α* and IKK*α*/*β*, upstream of NF-*κ*B, were also activated more in GADD34-deficient cells than in control cells by LPS (Figure 6b). Additionally, degradation of I*κ*B*α* was observed in GADD34-deficient cells after LPS stimulation (Figure 6b). However, the phosphorylation of TGF-*β* activated kinase 1 (TAK1), upstream of IKK*β*, showed no difference between GADD34-deficient and control cells (Figure 6b). In another experiment, peritoneal macrophages from GADD34KO mice also showed higher activation of NF-*κ*B signaling molecules, such as IKK*α*/*β*, I*κ*B*α*, and NF-*κ*B, than macrophages from WT mice (Supplementary Figure S5B). Next, we performed immunoprecipitation on RAW264.7 cells with an anti-GADD34 antibody and confirmed the binding of GADD34 to IKK*β* in macrophages after LPS stimulation (Figure 6d). Thus, GADD34 regulates the activation of IKK*β* in macrophages after LPS stimulation. To test whether the expression of IL-6 was the direct result of NF-*κ*B p65 binding to the IL-6 promoter, we performed chromatin immunoprecipitation (ChIP) experiments. As a result, GADD34-deficient cells showed much more NF-*κ*B p65 binding to the IL-6 promoter than did control cells (Figure 6e). Taken together, these data indicate that GADD34 inhibits the activation of TLR4-mediated NF-*κ*B signaling in macrophages through dephosphorylating IKK*β*, and suppresses pro-inflammatory cytokine production.

### GADD34 inhibits the production of inflammatory cytokines through suppressing TLR signaling in Kupffer cells

**Figure 7:**
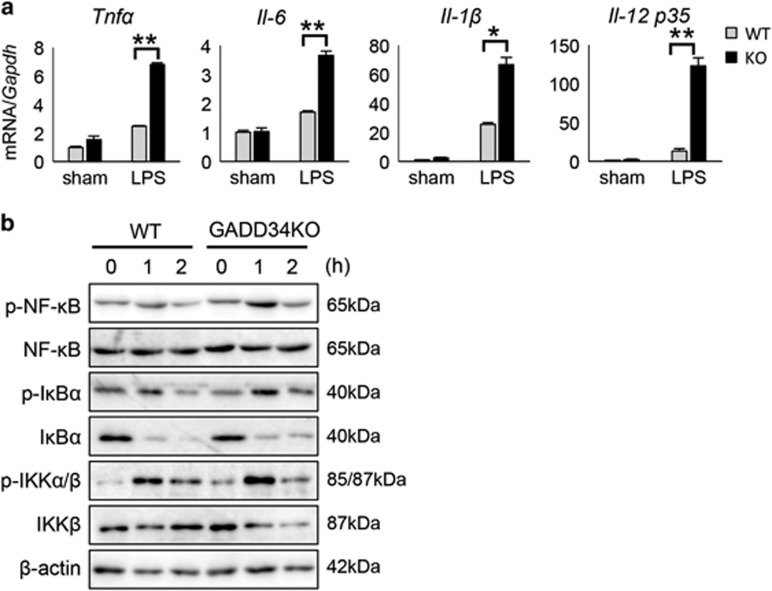
GADD34 regulates cytokine production from Kupffer cells by inhibiting TLR4-mediated NF-*κ*B signaling. Kupffer cells were isolated from WT and GADD34-deficient liver and treated with LPS (1 *μ*g/ml). (**a**) Real-time PCR analysis for inflammatory cytokine expression in Kupffer cells treated with LPS for 4 h. 100 ng total RNA of Kupffer cells was used for the synthesis of first-strand cDNA. Data represent means±S.E.M. (*n*=3). *P<0.05, **P<0.01. (**b**) Western blotting analysis of Kupffer cells treated with LPS for 0, 1, or 2 h. These data are representative of three separate experiments. The original data are presented in Supplementary Figure S10

We next examined whether GADD34 could regulate inflammatory responses following LPS stimulation in liver-resident Kupffer cells. Kupffer cells were isolated from the livers of WT and GADD34KO mice. Isolated Kupffer cells were stimulated with LPS. The expression of inflammatory cytokines such as *Tnfα*, *Il-6*, *Il-1β*, and *Il-12 p35* was significantly higher in GADD34KO Kupffer cells than in WT Kupffer cells after LPS stimulation (Figure 7a). Moreover, it was confirmed that the activation of the NF-*κ*B signaling pathway by LPS was higher in GADD34KO Kupffer cells than in WT Kupffer cells (Figure 7b). These results indicate that GADD34 inhibited the activation of TLR4-mediated NF-*κ*B signaling in macrophages, including Kupffer cells, and suppressed inflammatory cytokine production. Thus, our results clearly show that GADD34 has a critical role in suppressing the inflammatory responses of macrophages as well as tissue injury induced by bacterial infection.

## Discussion

In this report, we clearly demonstrated that GADD34 has a critical role in LPS-induced sepsis and tissue injury. Sepsis is a systemic inflammatory syndrome that results from a harmful or damaging host response to infection. Severe sepsis exhibits high lethality due to cell and tissue damage and multiple organ failure.^1^ High amounts of inflammatory cytokines in serum are known to be associated with patient lethality in sepsis.^1, 4, 5^ GADD34 markedly lowered the lethality of LPS-exposed mice in association with reduced serum levels of pro-inflammatory cytokines (Figures 1a and b). Additionally, GADD34 reduced serum IL-10 concentrations, which are normally increased by LPS stimulation (Figure 1b). Generally speaking, anti-inflammatory cytokines, including IL-10, control and downregulate the inflammatory response. However, in sepsis, it has been shown that anti-inflammatory cytokines such as IL-10 are significantly increased in the circulation of patients, and are regarded as important inflammatory markers.^5^ In this LPS-induced murine sepsis model, the levels of IL-10 in serum correlated with the severity of sepsis as well as pro-inflammatory cytokines. Pro-inflammatory cytokines, such as TNF*α*, promote liver destruction and acute liver failure.^27^ We confirmed that GADD34 reduced pro-inflammatory cytokine production in the liver, and hepatic destruction, as shown by decreased AST and ALT levels in serum.

The ER stress response has been linked to several liver diseases, such as acute liver injury, obesity-associated fatty liver, and viral hepatitis.^28, 29, 30^ Previous studies from our group and others have reported that GADD34, which is induced by ER stress, dephosphorylated the translational initiation factor eIF2*α* and recovered from the shut-off of translation.^25, 26^ Recently, Rao *et al.*^30^ documented that CHOP, which is downstream of eIF2*α*, promotes liver injury and hepatic apoptosis in a GalN/LPS-induced acute liver failure model. In our study, we showed that the expression of p-eIF2*α*, ATF4, and CHOP was markedly upregulated, and more apoptotic cells were detected in LPS-treated livers from GADD34KO mice compared with WT mice. Collectively, GADD34 inhibited hepatic apoptosis through downregulating eIF2*α*-ATF4-CHOP pathway induced by LPS (Figure 3).

GADD34 is linked to cytokine production in response to infection. TLR3 ligands, such as poly(I:C), induce GADD34 to promote cytokine production, including IFN-*β* and IL-6, through eIF2*α* dephosphorylation.^23, 24^ In this study, we also observed that GADD34-deficient macrophages secreted lower levels of pro-inflammatory cytokines than control cells by following poly(I:C) stimulation (Supplementary Figure S4). However, when we stimulated macrophages with individual TLR1–9 ligands, most of the TLR ligands, including TLR4 ligands, induced higher pro-inflammatory cytokine production in GADD34-deficient cells than in control cells (Supplementary Figure S4, Figures 5b and c). LPS stimulation did not affect the phosphorylation of eIF2*α* (Figure 6a). Our data reveal that GADD34 has a different role beyond regulating ER stress responses, including regulating inflammatory responses against bacterial infection.

NF-*κ*B signaling enhances the mRNA expression of pro-inflammatory cytokines after LPS stimulation. A previous report showed that CUEDC2 interacted with GADD34 and suppressed the activation of IKK, which is the upstream kinase of NF-*κ*B, and that in turn suppressed pro-inflammatory cytokine production.^11^ Our study supports these previous results, and demonstrates that GADD34 is able to suppress sepsis, tissue injury, and macrophage activation after LPS stimulation through dephosphorylation of IKK*β* in macrophages.

In conclusion, we revealed that GADD34 attenuates LPS-induced lethality and acute liver injury through suppressing macrophage activation. Furthermore, our study demonstrated an anti-inflammatory role for GADD34 by repressing NF-*κ*B signaling in macrophages following LPS stimulation. Our findings indicate that GADD34 may be a novel therapeutic target for regulating inflammatory disorders.

## Materials and Methods

### Mice

All experiments used 6- to 8-week-old GADD34 (ppp1r15a) knockout (KO) mice and littermate wild-type (WT) mice. GADD34KO mice were purchased from MMRRC. GADD34KO mice were mated with C57BL/6 and backcrossed for more than 12 generations. These mice were maintained in the Animal Research Facility at Nagoya University Graduate School of Medicine under specific pathogen-free conditions. This work was approved by the ethical committee of Nagoya University.

### Induction of LPS-induced sepsis

For survival studies, mice were intraperitoneally injected with LPS (Sigma, St. Louis, MO, USA) at 30 mg/kg body weight. Their survival was monitored until 40 h after LPS injection. For inflammatory analysis, mice were injected with LPS at 5 mg/kg body weight. These mice were killed at 0 h (sham), 4 h, and 16 h after LPS injection, and their serum, liver, and kidneys were harvested.

### Induction of LPS-induced acute lung failure

Mice were treated with LPS (30 or 60 *μ*g/mouse) by transnasal administration. At 16 h after LPS treatment, lung tissue was harvested from these mice and analyzed by H&E staining or real-time PCR analysis, as described below.

### Cell culture

Murine macrophage RAW264.7 cells were obtained from RIKEN. RAW264.7 cells were cultured in DMEM (Sigma) supplemented with 10% heat-inactivated FBS (Equitech-Bio Inc., Kerrville, TX, USA). The translation of GADD34 mRNA in RAW264.7 cells was knocked down (shGADD34) as previously described.^22^ Non-target control shRNA (Sigma) was used as a control (shControl). Human monocytic THP-1 cells were obtained from RIKEN. THP-1 cells were cultured in RPMI-1640 (Sigma) supplemented with 10% heat-inactivated FBS (Hyclone, Logan, UT, USA). THP-1 was transfected with 10 nM siRNA using Lipofectamine RNAiMax (Invitrogen, Waltham, MA, USA) according to the manufacturer's instructions. siRNAs were obtained from Ambion (Waltham, MA, USA). The sequence of siRNA used to knockdown GADD34 is 5′-GGAUCAGCCCGAGGAUGAAA-3′. Non-targeted control siRNA was used as a control. Recombinant experiments were approved by the Committee of Nagoya University Graduate School of Medicine.

### Isolation of Kupffer cells from liver

Kupffer cells were isolated according to the method reported by Li *et al.*^31^ with some modifications. In brief, mice were anesthetized by injection of avertin peritoneally. The liver was perfused *in situ* with 20 ml HBSS (Gibco, Waltham, MA, USA) 37 °C via the portal vein. The liver was then excised and minced into small pieces. The liver tissues were dispersed in RPMI-1640 (Sigma) containing 0.1% collagenase I (Wako, Osaka, Japan) and incubated under continuous agitation at 37 °C for 30 min. Following digestion, the liver homogenate was filtered through a nylon filter (100 *μ*m pore size) to remove undigested tissue; and the cell suspension was centrifuged at 300 × *g* for 5 min at 4 °C. The cell sediment was resuspended with 10 ml RPMI-1640 and centrifuged at 300 × *g* for 5 min at 4 °C. Next, the cell sediments were resuspended with 10 ml RPMI-1640 and centrifuged at 50 × *g* for 3 min at 4 °C. The top aqueous phase was transferred into a new centrifuge tube and centrifuged at 300 × *g* for 5 min at 4 °C. To purify the obtained cell population, the cell sediments were seeded into a 35-mm diameter culture dish at a density of 3–5 × 10^6^/dish in DMEM supplemented with 10% heat-inactivated FBS (Gibco) and penicillin/streptomycin (Invitrogen). After incubation for 2 h at 37 °C, non-adherent cells were removed from the dish by gently washing with pre-warmed PBS, and the adherent cells were used as Kupffer cells. Isolated Kupffer cells were identified by microscopic observation and flow-cytometric analysis. More than 80% of the attached cells were F4/80-positive macrophages (Supplementary Figures S6A and B).

### Isolation of peritoneal macrophages

In all, 3% thioglycolate (1 ml/mouse; Sigma) was intraperitoneally injected into mice. At 3 days after injection, murine peritoneal macrophages were collected from the peritoneal cavity. The cells were washed with ice-cold PBS and cultured in RPMI-1640 (Sigma) supplemented with 10% heat-inactivated FBS (Equitech-Bio Inc.), 50 *μ*M 2-mercaptoethanol and penicillin/streptomycin (Invitrogen). After culture for 1 h, 3 × 10^6^ attached cells were stimulated with LPS (1 *μ*g/ml; Sigma) in a 6-well cell culture plate. For analysis, supernatants and protein samples of these cells were collected at the indicated time point (0–8 h) after LPS stimulation.

### ELISA

Serum samples and supernatants of cultured cells were collected. TNF*α*, IL-6, IL-1*β*, IL-12, MIP-2, and IL-10 proteins in serum and in cultured supernatants were determined by specific ELISA kits (R&D Systems, Minneapolis, MN, USA) according to the manufacturer's instructions.

### Histopathological analysis

Murine tissue samples were fixed in 4% paraformaldehyde neutral buffer solution for paraffin embedding. Paraffin-embedded tissues were cut into 4-*μ*m sections and stained with hematoxylin and eosin (H&E). Histology scores for livers were assigned as follows, based on the severity of necrosis, bleeding, and infiltration in the liver. *Necrosis*: normal=0, mild (focal piecemeal necrosis)=1, moderate (continuous necrosis in <50% of focal areas)=2, and severe (continuous necrosis in >50% in focal areas)=3. *Bleeding*: normal=0, mild (<30% of focal areas)=1, moderate (30–50% of focal areas)=2, and severe (>50 of focal areas)=3. *Infiltration*: normal=0, mild (2- to 3-fold inflammatory cells)=1, moderate (3- to 10-fold inflammatory cells)=2, and severe (>10-fold inflammatory cells)=3.

### Biochemical assays

AST and ALT levels in serum were determined using the AST Reagent kit and ALT Reagent kit (Wako) with a microplate reader (BioTek, Winooski, VT, USA).

### TUNEL staining and immunohistochemistry

For detecting apoptosis, paraffin-embedded slides were deparaffinized and TUNEL staining was performed using an In Situ Apoptosis Detection Kit (Takara, Shiga, Japan). After 3 min counterstaining with 4,6-diamidino-2-phenylindole (DAPI), the TUNEL-stained slides were analyzed with a Nikon A1RSi Laser Scanning Confocal Microscope (Nikon, Tokyo, Japan). For immunohistochemistry, paraffin-embedded slides were deparaffinized, and boiled in citrate buffer for antigen retrieval and stained overnight with anti-F4/80 antibodies (BMA, Augst, Switzerland; 1 : 200 dilution) or anti-CyclinD1 antibodies (Santa Cruz, Santa Cruz, CA, USA; 1 : 200 dilution).

### Western blot analysis

Tissue samples were homogenized in standard RIPA buffer with PMSF. Protein concentrations were quantified with the Lowry assay using the DC protein assay kit (Bio-Rad, Hercules, CA, USA). Then, 30 *μ*g of total protein was diluted to the same volume 2 × SDS-sample buffer (62.5 mM Tris-HCl pH 6.8, 2% SDS, 20% glycerol, 5% 2-mercaptoethanol, and 0.025% bromophenol blue). Cultured cells were lysed in 2 × SDS-sample buffer. Protein lysates were separated by 6–10% SDS-gel electrophoresis, and transferred onto Immobilon-P membranes (Millipore, Billerica, MA, USA). Membranes were blocked in PBST buffer containing 3% skim milk for 1 h at room temperature, probed with primary antibodies and secondary HRP-conjugated antibodies (GE Healthcare, Little Chalfont, UK), and developed using ECL western blot detection reagent (GE Healthcare). Antibodies used in this study are as follows: anti-GADD34 was from Santa Cruz; anti-*β*-actin was from Sigma; and anti-p-eIF2*α*, anti-eIF2*α*, anti-ATF4, anti-CHOP, anti-p-NF-*κ*B p65, anti-NF-*κ*B p65, anti-p-I*κ*B*α*, anti-I*κ*B*α*, anti-p-IKK*α*/*β*, anti-IKK*β*, anti-p-TAK1, anti-p-IRF3, anti-IRF3, anti-p-IKKɛ, and anti-IKKɛ were from Cell Signaling Technology (Danvers, MA, USA).

### RT-PCR analysis

Total RNA of cultured cells was isolated using RNeasy mini kits (Qiagen, Hilden, Germany), and tissue RNA was isolated using TRIzol (Invitrogen) according to the manufacturer's recommended protocol. Residual genomic DNA was digested and removed using DNase I (Invitrogen) treatment. Unless otherwise specified, first-strand cDNA was synthesized using 1 *μ*g total RNA and a High Capacity cDNA Reverse Transcription Kits (Applied BioSystems, Waltham, MA, USA) for RT-PCR. RT-PCR was performed using the Takara EX Taq (Takara) according to the manufacturer's instructions. Real-time PCR was performed using SYBR green (Toyobo, Osaka, Japan) according to the manufacturer's instructions. Expression data were normalized to *Gapdh* mRNA expression. For human THP-1 cells, expression data were normalized to *β-actin* mRNA expression. The primer sequences are shown in Supplementary Table S1.

### Flow-cytometric analysis

Liver tissues were homogenized to give single cell preparations. The cells were washed twice and 1 × 10^6^ cells were suspended in 50 *μ*l PBS supplemented with 1% FBS and stained for 20 min at 4 °C with directly conjugated fluorescent antibodies (1 : 500). Antibodies were as follows: eFluor450-conjugated anti-CD11b and allophycocyanin (APC)-conjugated anti-F4/80 were from eBioscience (San Diego, CA, USA). Stained cells were analyzed with an FACSCanto flow cytometer using FACSDiva software (BD Biosciences, Franklin Lakes, NJ, USA), and the data were analyzed with FlowJo software (TreeStar, Ashland, OR, USA).

### Immunofluorescence

Liver tissues were embedded in OCT compound (Sakura Finetek). OCT compound-embedded tissues were cut into 5-*μ*m sections and fixed in 4% paraformaldehyde. After rinsing with PBS, sections were permeabilized and treated with blocking buffer (0.2% Triton X-100, 0.2% bovine serum albumin (BSA), and 0.1% normal goat serum in PBS). After the blocking process, sections were incubated with FITC-conjugated anti-TNF*α* antibody (1 : 200 dilution; eBioscience), FITC-conjugated anti-IL-6 antibody (1 : 200 dilution; eBioscience), and APC-conjugated anti-F4/80 antibody (1 : 1000 dilution; eBioscience) in blocking buffer at 4 °C overnight. After that, sections were washed with PBS and incubated with DAPI for 5 min. After rinsing with PBS, the sections were mounted with mounting fluid and visualized under an A1Rsi inverted Confocal Microscope (Nikon).

### Immunoprecipitation assay

Cultured cells were lysed in RIPA buffer (25 mM Tris-HCl pH 8.0, 150 mM NaCl, 10% glycerol, 2 mM EDTA, 5 mM MgCl_2_, 0.5% NP-40, 5 mM NaF, 1 mM Na_3_VO_4_ containing protease inhibitors; Roche, Basel, Switzerland). After 20 min on ice, lysates were centrifuged for 10 min at 10 000 × *g* and 4 °C to remove debris. Cell lysates were incubated overnight at 4 °C with antibodies and protein G Sepharose (GE Healthcare). Sepharose samples were centrifuged, washed three times with RIPA buffer and one time with 0.5 M Tris-HCl pH 8.0 and boiled for 3 min with SDS-sample buffer. Antibody to IKK*β* was obtained from Cell Signaling Technology. Anti-GADD34 was from Santa Cruz. Anti-rabbit IgG was from Sigma.

### ChIP assay

ChIP assays were performed using Chromatin immunoprecipitation kits (Millipore) according to the manufacturer's recommended protocol. Antibodies used for ChIP were follows: anti-NF-*κ*B p65 antibody (Cell Signaling Technology), anti-rabbit IgG (Sigma), and anti-RNA polymerase II (Millipore). The primer sequences are shown in Supplementary Table S1.

### Statistical analysis

Data are expressed as means±standard error of the mean (S.E.M.). Differences were analyzed by Student's *t*-test. The survival curves were estimated according to the method of Kaplan–Meier and compared with the generalized Wilcoxon test. *P-*values less than 0.05 were considered as statistically significant.

## Abbreviations

GADD34: Growth arrest and DNA damage inducible protein 34
WT: wild type
KO: knockout
ER: endoplasmic reticulum
eIF2*α*: eukaryotic initiation factor 2*α*
LPS: lipopolysaccharide
TLR: Toll-like receptor
NF-*κ*B: nuclear factor-*κ*B
IKK: I*κ*B kinase
I*κ*B: inhibitor of NF-*κ*B
TAK1: TGF-*β* activated kinase 1
IRF3: interferon-regulatory factor 3
TNF*α*: tumor necrosis factor-*α*
IL-6: interleukin-6
IL-1*β*: interleukin-1*β*
IL-12: interleukin-12
IL-10: interleukin-10
MIP-2: macrophage inflammatory protein-2
IFN-*β*: interferon-*β*
AST: aspartate aminotransferase
ALT: alanine aminotransferase
PERK: protein kinase RNA-like endoplasmic reticulum kinase
ATF4: activating transcription factor 4
CHOP: C/EBP homologous protein
