# Supplementary material for: GADD34 suppresses lipopolysaccharide-induced sepsis and tissue injury through the regulation of macrophage activation
Source: Cell Death Dis. 2016 May 12;7(5):e2219–. doi: 10.1038/cddis.2016.116 (PMC4917654; doi:10.1038/cddis.2016.116)
Supplement: Supplementary Figures [file cddis2016116x1.docx]

Supplementary Table 1. The following primers were used for RT-PCR

|  |  |  |
| --- | --- | --- |
| genes | sequences (Forward ; 5'-3') | sequences (Reverse ; 5'-3') |
| Mouse *Atf4* | TCCTGAACAGCGAAGTGTTG | ACCCATGAGGTTTCAAGTGC |
| Mouse *Chop* | GCATGAAGGAGAAGGAGCAG | CTTCCGGAGAGACAGACAGG |
| Mouse *Perk* | GACAGACTGCGGAGACAACA | GACCGGGTATAGGGAGAAGC |
| Mouse *Tnfα* | GCCCATATACCTGGGAGGAG | CACCCATTCCCTTCACAGAG |
| Mouse *Il-6* | CCGGAGAGGAGACTTCACAG | TCCACGATTTCCCAGAGAAC |
| Mouse *IL-1β* | GCCCATCCTCTGTGACTCAT | AAGGCCACAGGTATTTTGTCG |
| Mouse *IL-12 p35* | catcgatgagctgatgcagt | cagatagcccatcaccctgt |
| Mouse *IL-12 p40* | AGGTCACACTGGACCAAAGG | TGGTTTGATGATGTCCCTGA |
| Mouse *Mip-2* | TCCAGAGCTTGAGTGTGACG | AGGCACATCAGGTACGATCC |
| Mouse *IFN-β* | ccctatggagatgacggaga | ctgtctgctggtggagttca |
| Mouse *Gapdh* | AACTTTGGCATTGTGGAAGG | ACACATTGGGGGTAGGAACA |
| Human *Gadd34* | GAGGAGGCTGAAGACAGTGG | AATTGACTTCCCTGCCCTCT |
| Human *Il-6* | AGGAGACTTGCCTGGTGAAA | CAGGGGTGGTTATTGCATCT |
| Human *β-actin* | GATGAGATTGGCATGGCTTT | GTCACCTTCACCGTTCCAGT |
| Mouse *Il-6promoter κB site* | CGATGCTAAACGACGTCACATTGTGCA | CTCCAGAGCAGAATGAGCTACAGACAT |
|  |  |  |
