# Supplementary material for: GADD34 suppresses lipopolysaccharide-induced sepsis and tissue injury through the regulation of macrophage activation
Source: Cell Death Dis. 2016 May 12;7(5):e2219–. doi: 10.1038/cddis.2016.116 (PMC4917654; doi:10.1038/cddis.2016.116)
Supplement: Supplementary Table 1 [file cddis2016116x2.docx]

**GADD34 suppresses lipopolysaccharide-induced sepsis and tissue injury through the regulation of macrophage activation**

**Supplementary Figure Legends**

**Supplementary Figure S7.** The original immunoblot images for Figure 3 and 5.

**Supplementary Figure S8.** The original immunoblot images for Figure 6A and 6B.

**Supplementary Figure S9.** The original immunoblot images for Figure 6C and 6D.

**Supplementary Figure S10.** The original immunoblot images for Figure 6D, 6E and 7.

**Supplementary Figure S11.** The original immunoblot images for Supplementary Figure S4 and S5.

**Supplementary Figure S12.** The original immunoblot images for Supplementary Figure S5.
