# Supplementary figures and images for: GADD34 suppresses lipopolysaccharide-induced sepsis and tissue injury through the regulation of macrophage activation
Source: Cell Death Dis. 2016 May 12;7(5):e2219–. doi: 10.1038/cddis.2016.116 (PMC4917654; doi:10.1038/cddis.2016.116)

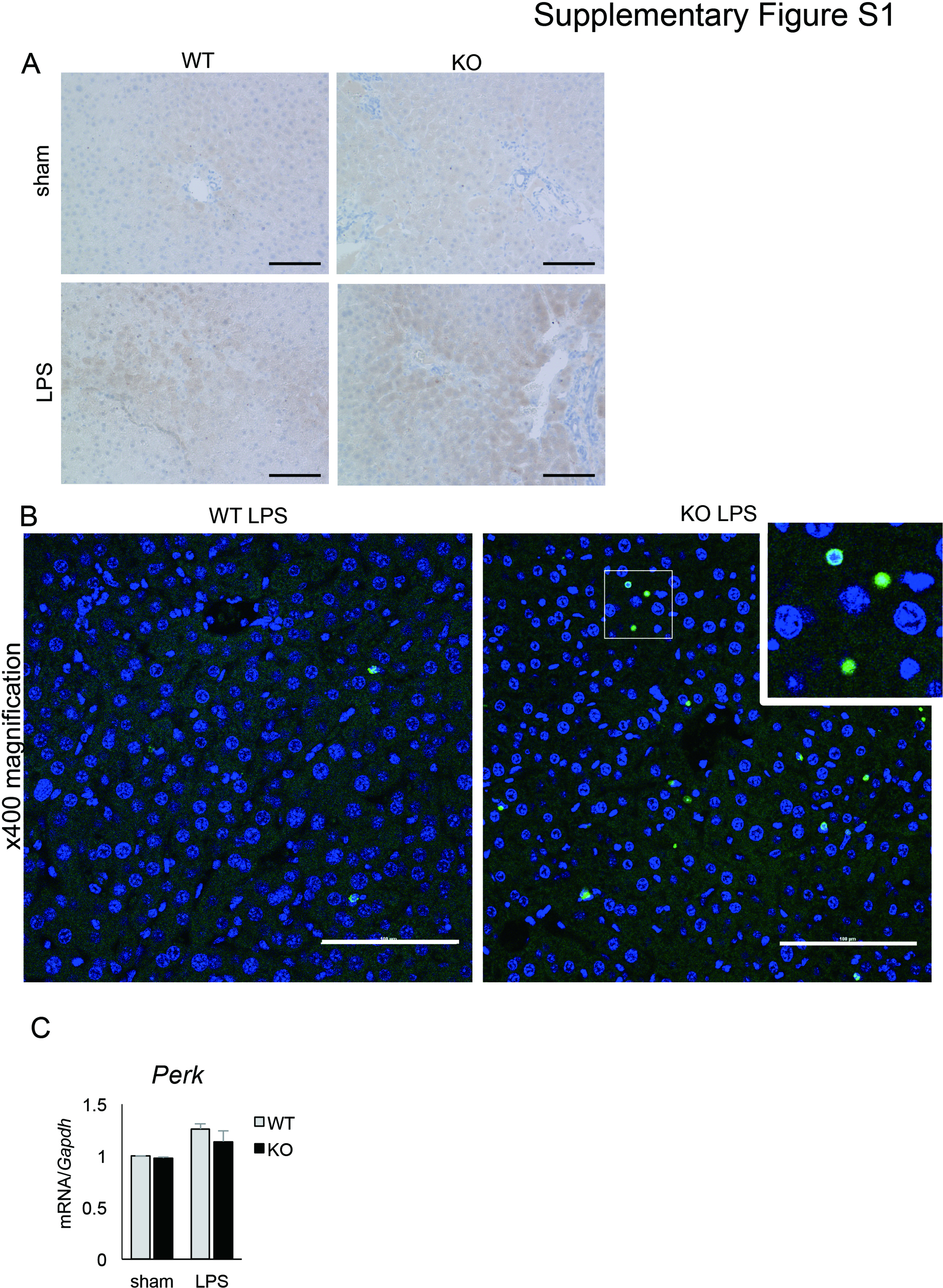

Supplement: Supplementary Figure S1 [file cddis2016116x3.tif]

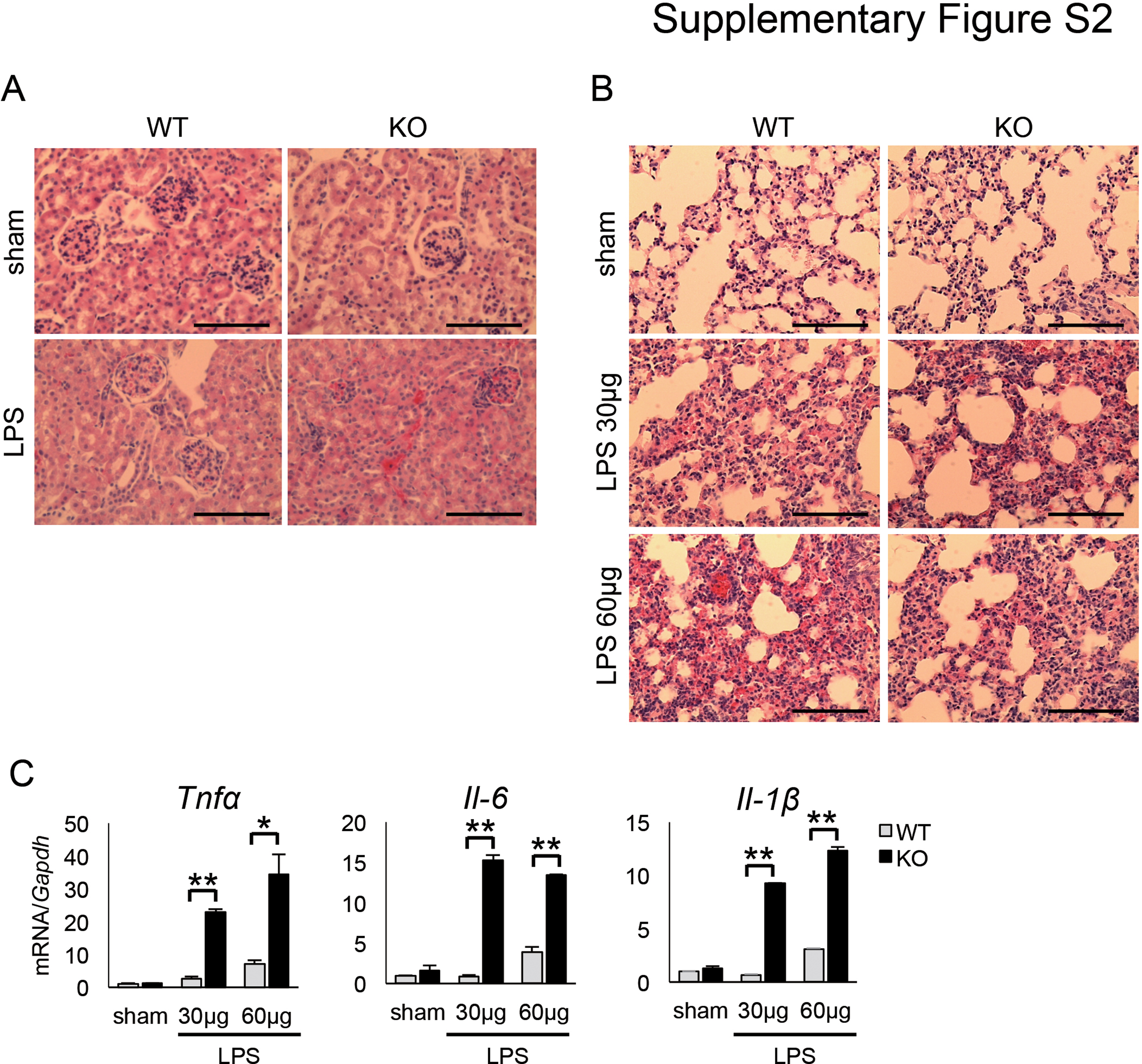

Supplement: Supplementary Figure S2 [file cddis2016116x4.tif]

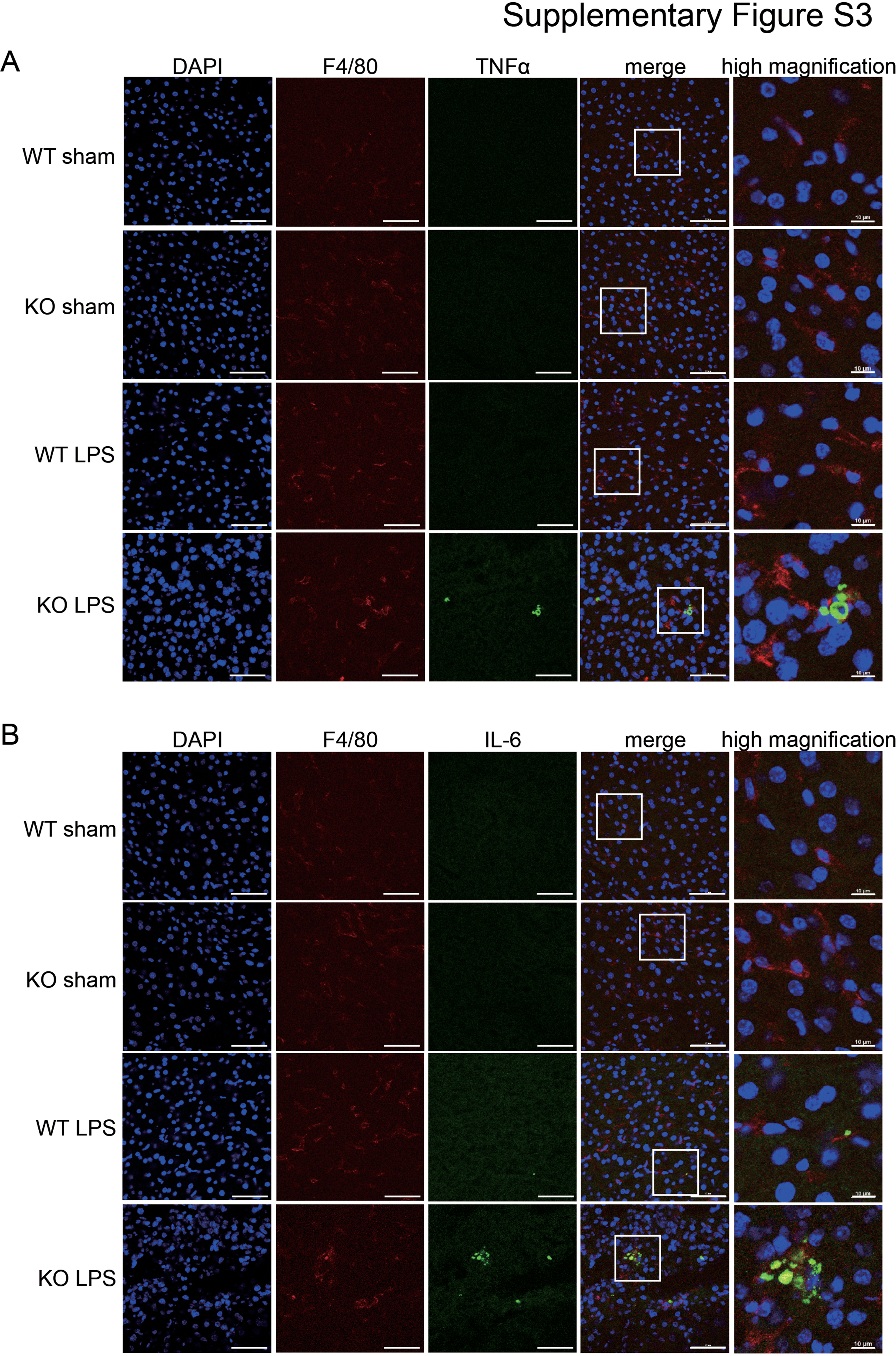

Supplement: Supplementary Figure S3a [file cddis2016116x5.tif]

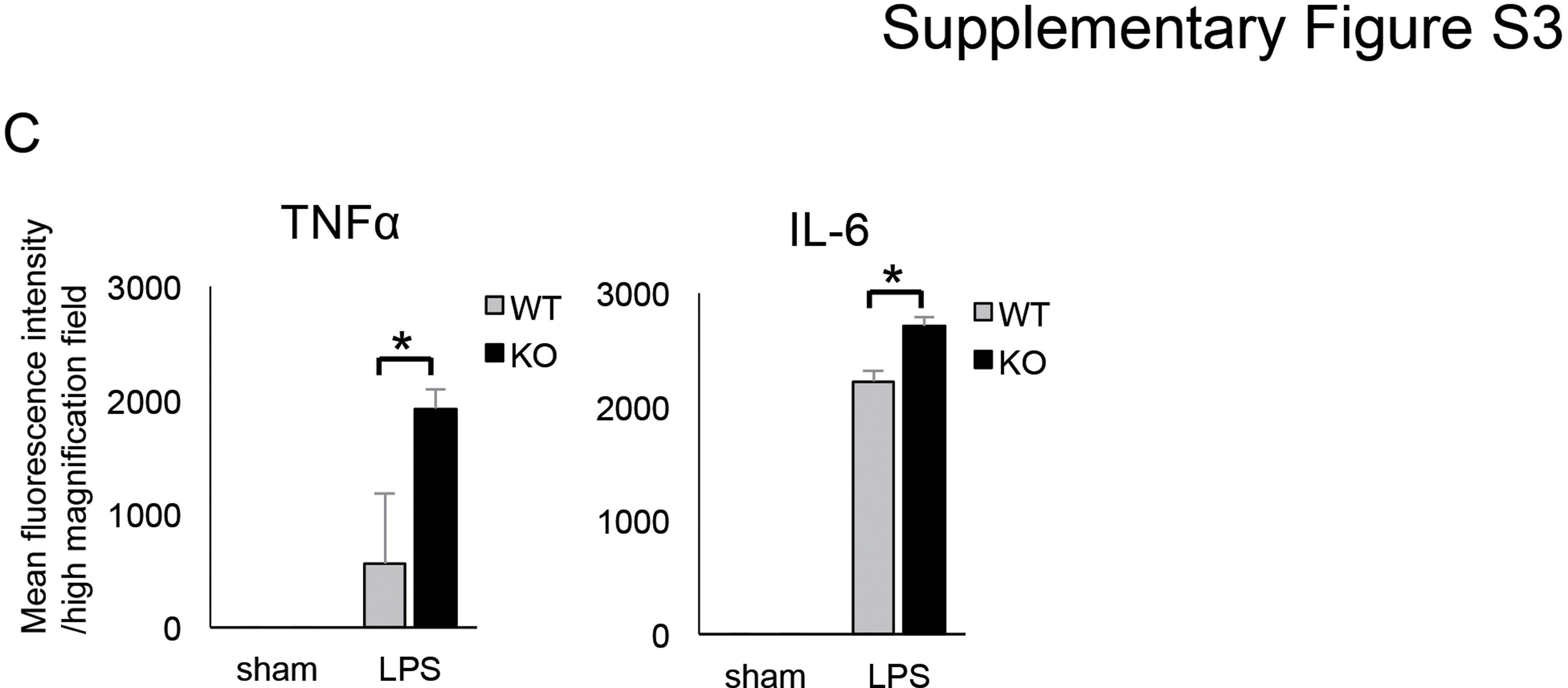

Supplement: Supplementary Figure S3b [file cddis2016116x6.tif]

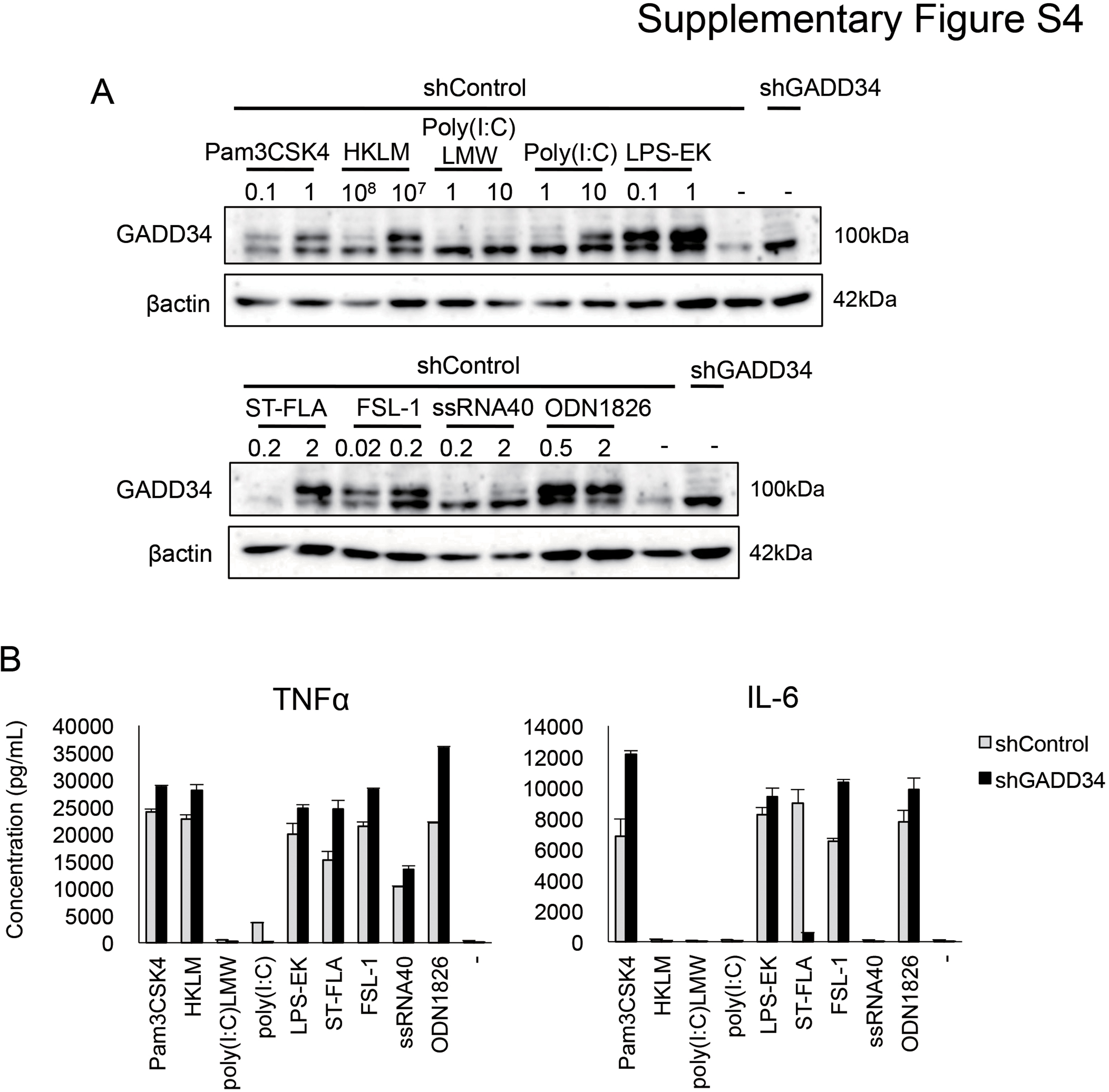

Supplement: Supplementary Figure S4 [file cddis2016116x7.tif]

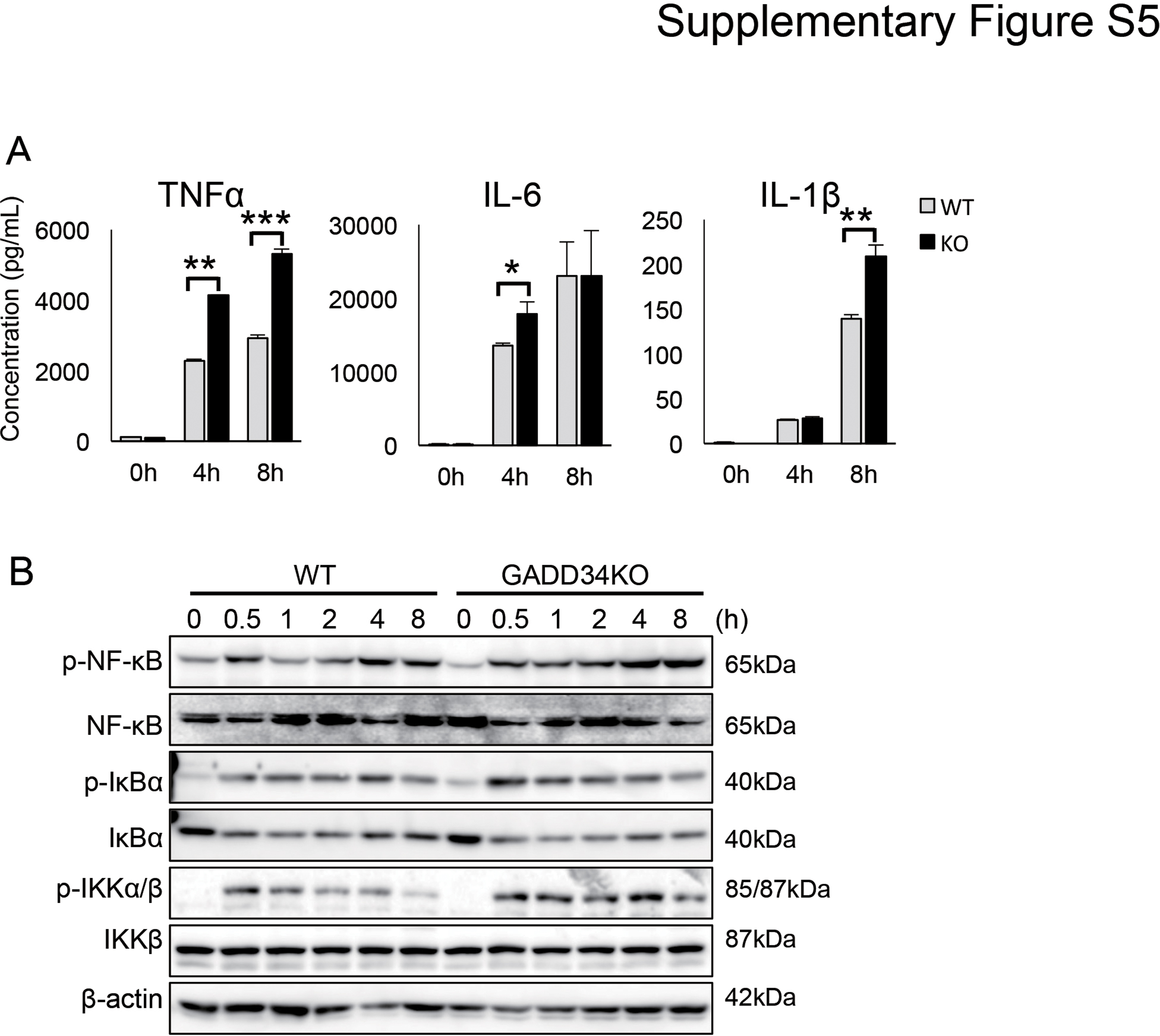

Supplement: Supplementary Figure S5 [file cddis2016116x8.tif]

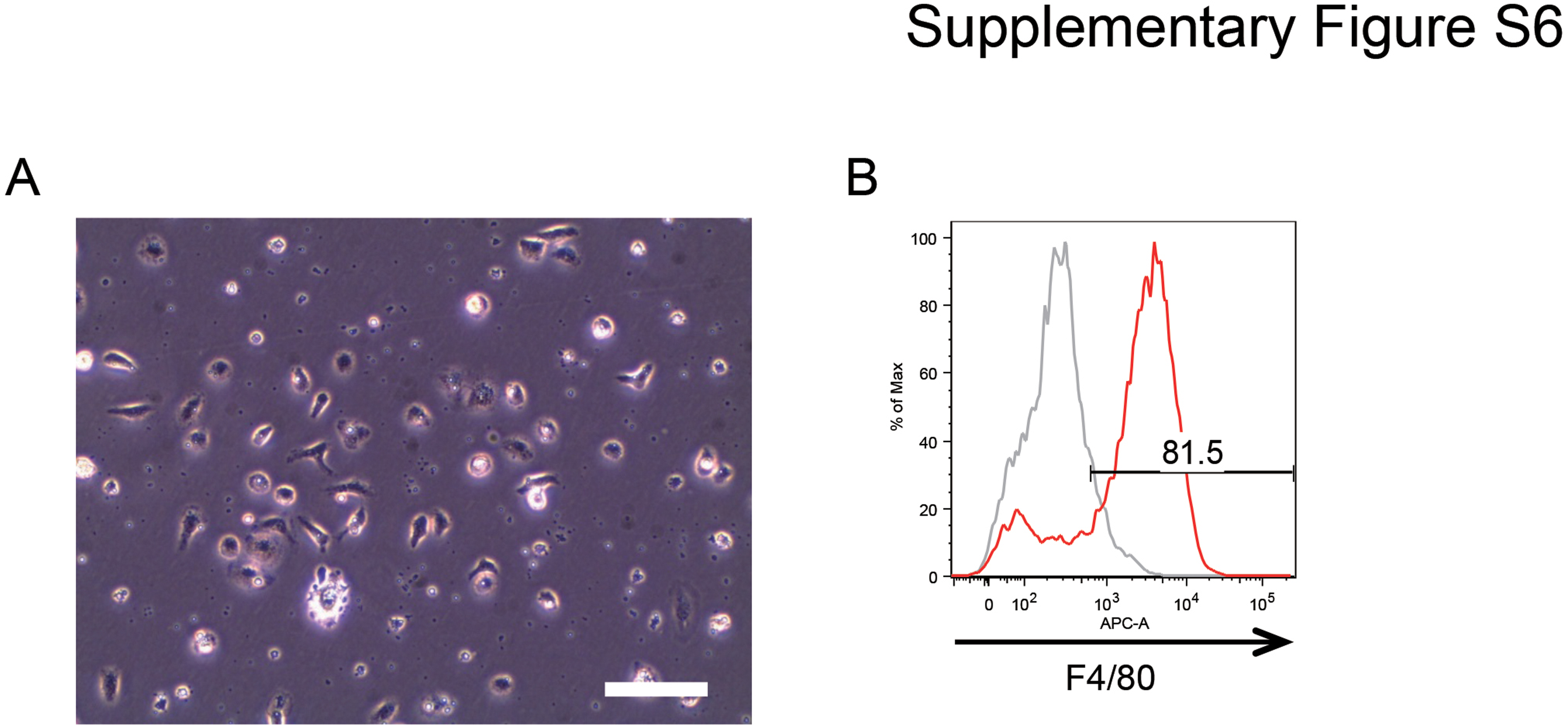

Supplement: Supplementary Figure S6 [file cddis2016116x9.tif]

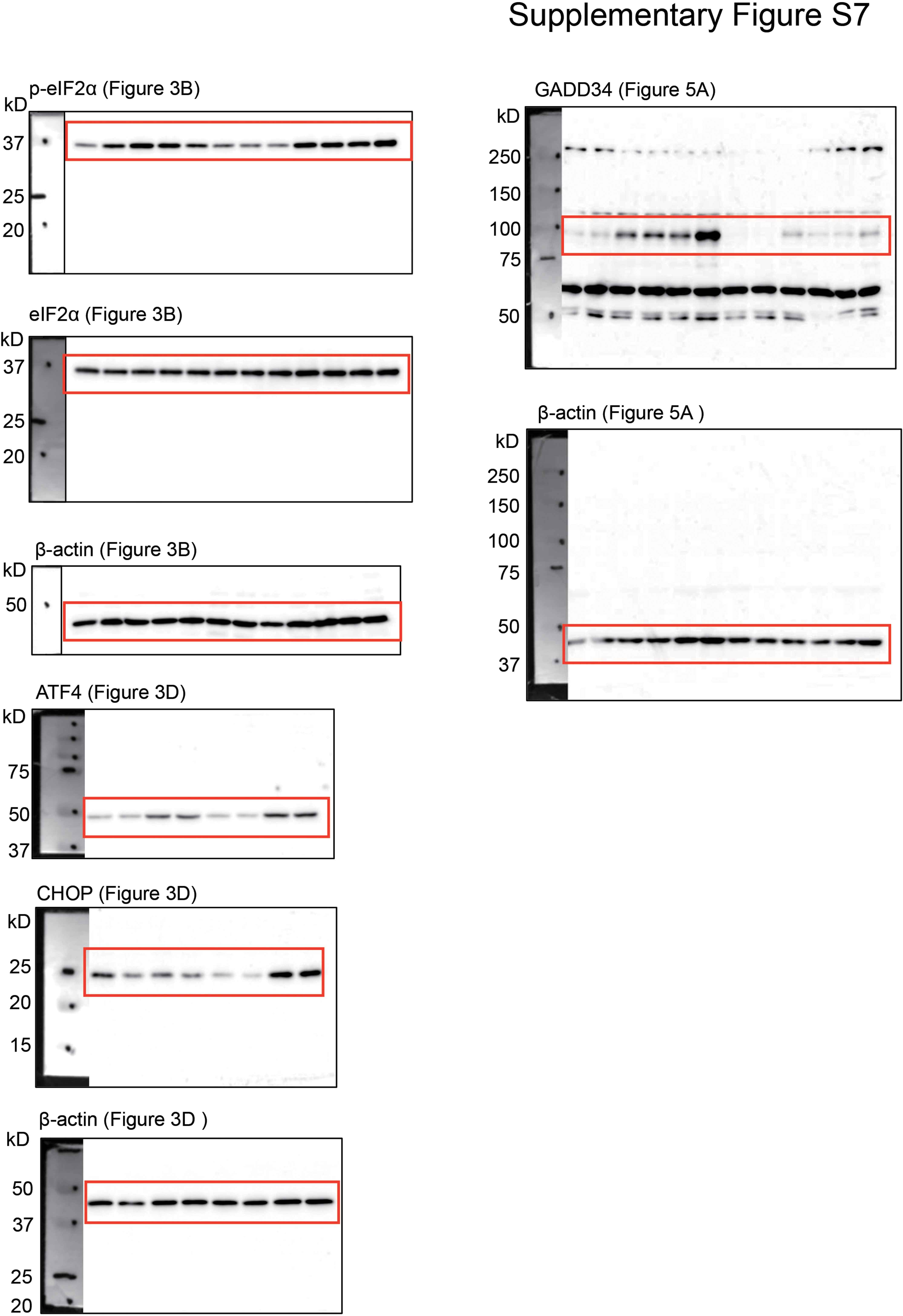

Supplement: Supplementary Figure S7 [file cddis2016116x10.tif]

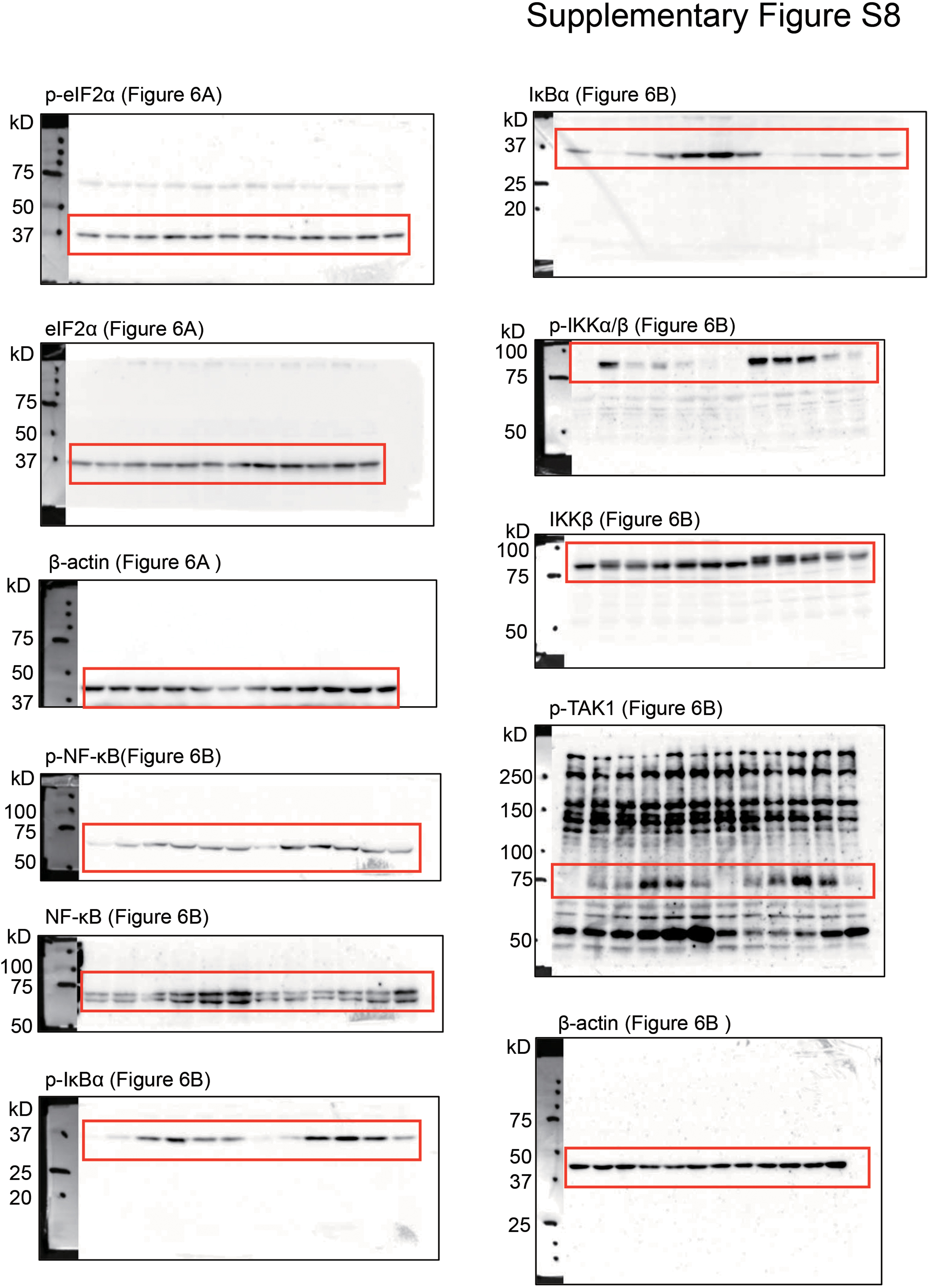

Supplement: Supplementary Figure S8 [file cddis2016116x11.tif]

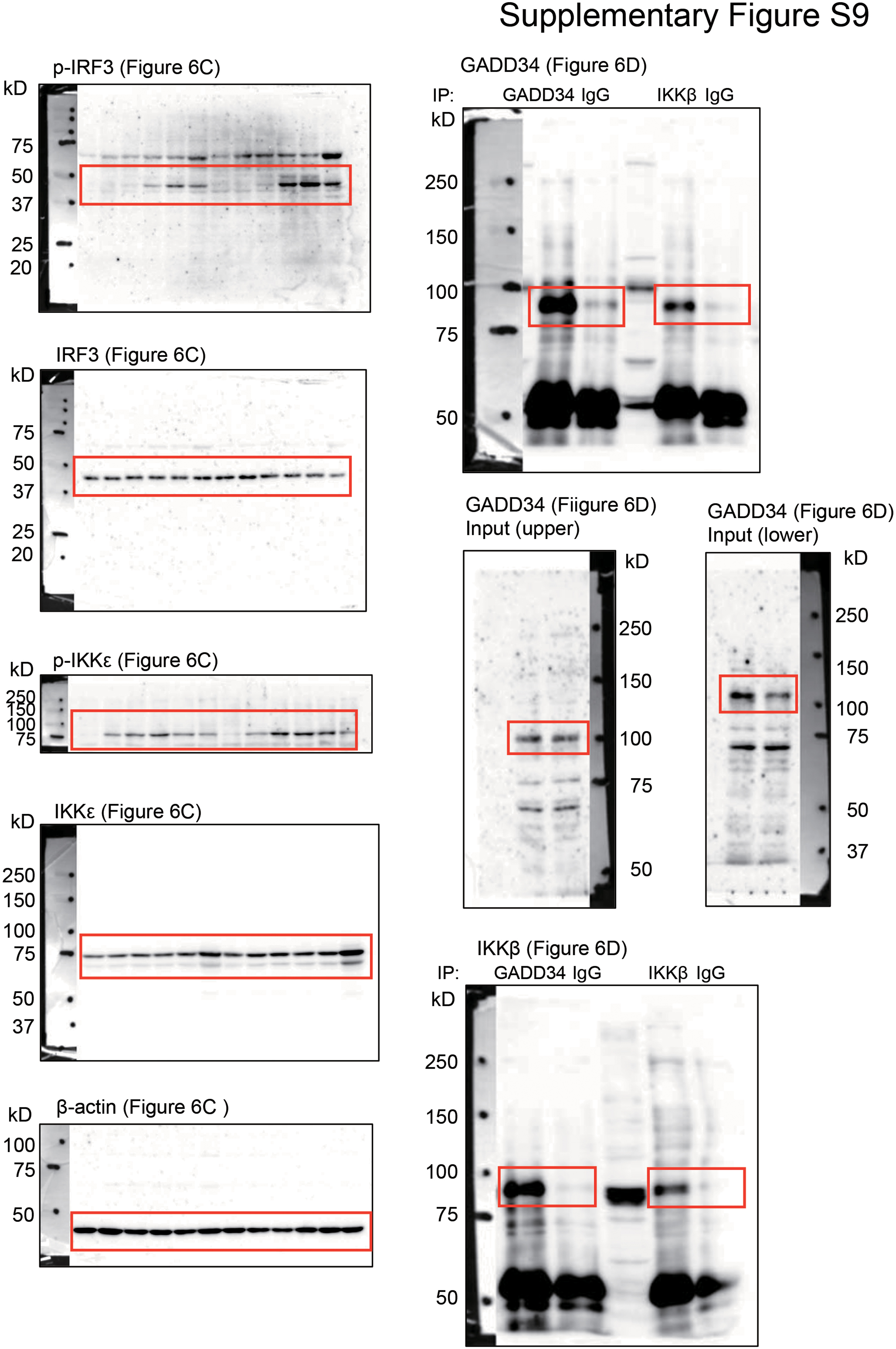

Supplement: Supplementary Figure S9 [file cddis2016116x12.tif]

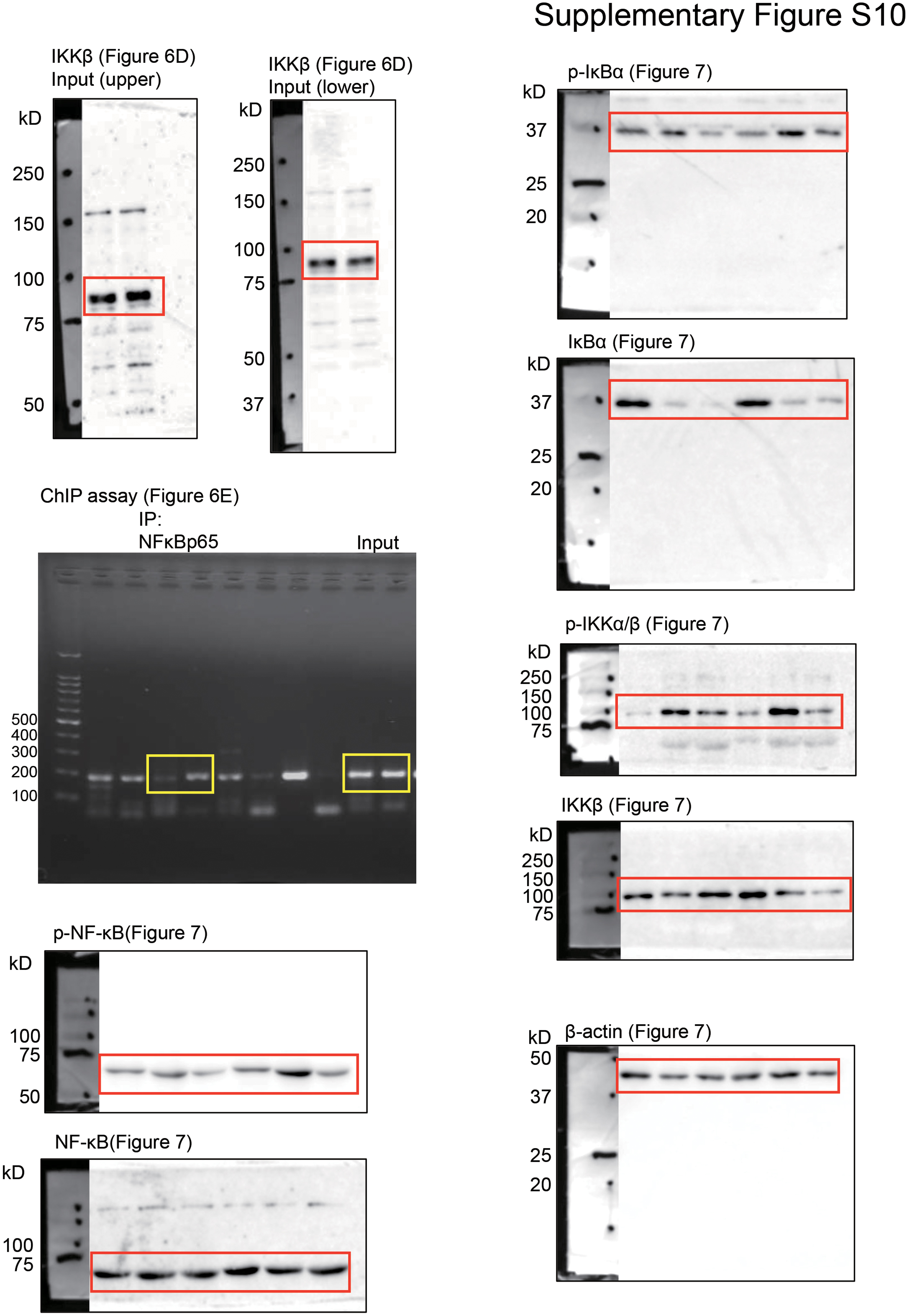

Supplement: Supplementary Figure S10 [file cddis2016116x13.tif]

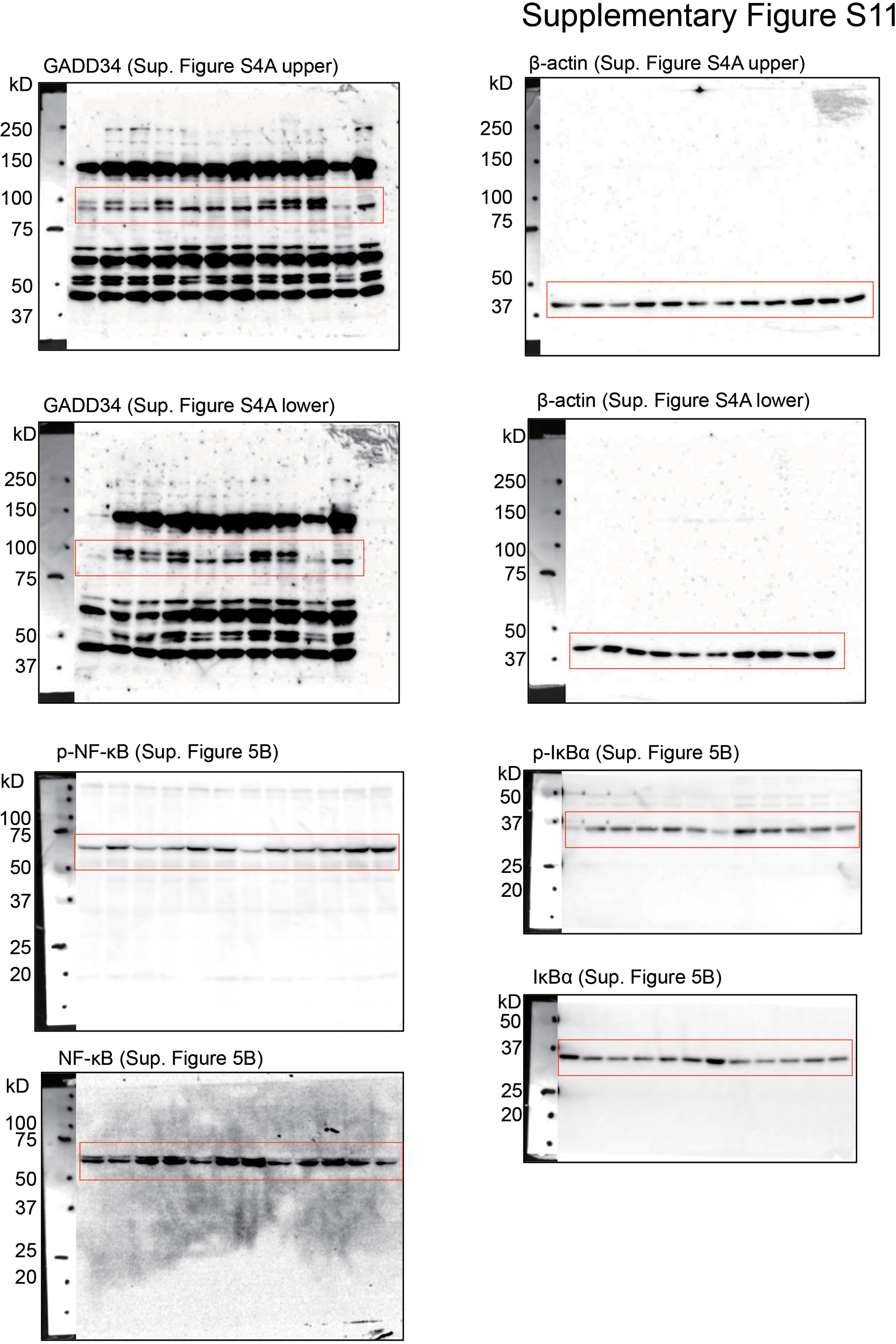

Supplement: Supplementary Figure S11 [file cddis2016116x14.tif]

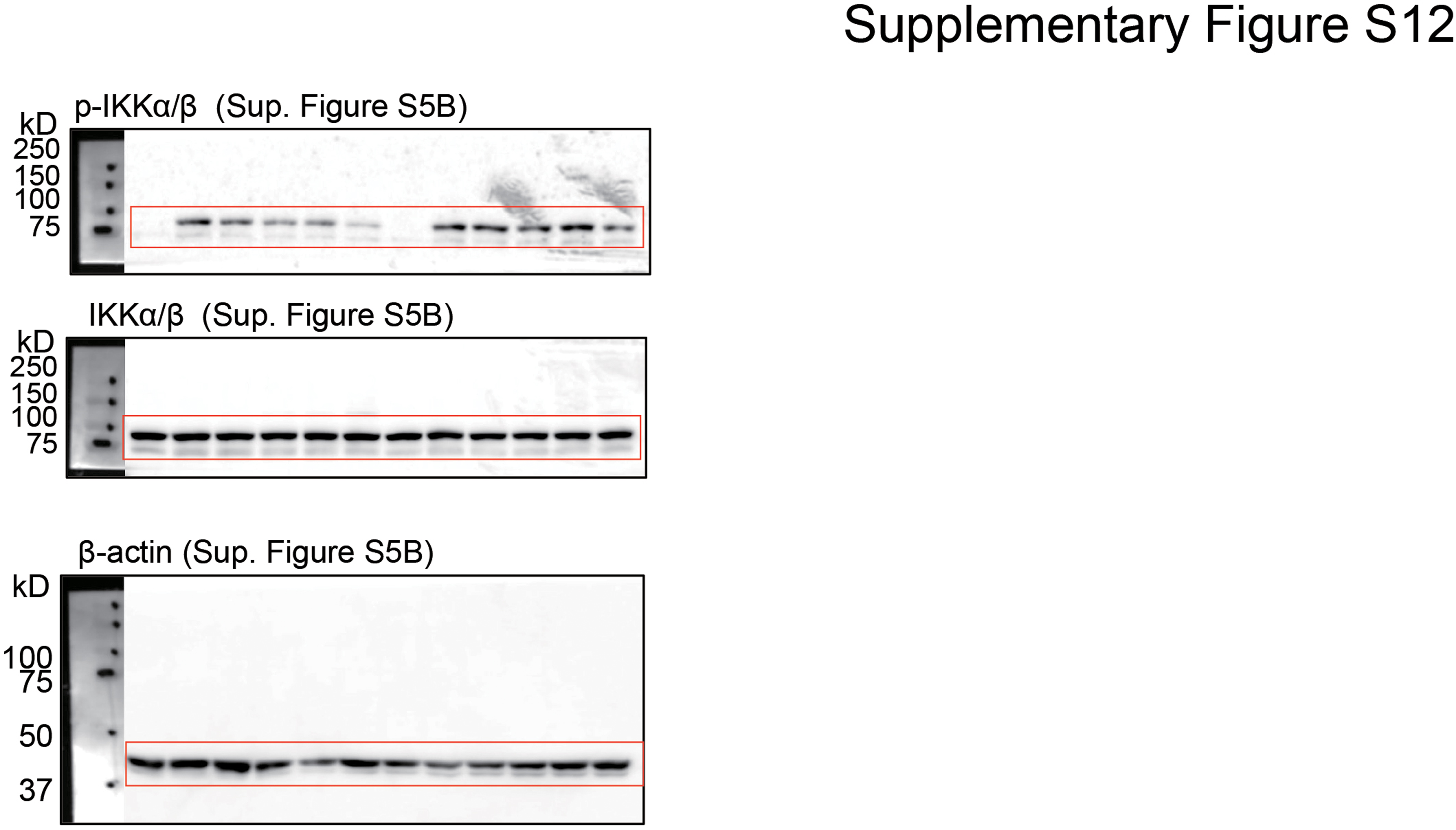

Supplement: Supplementary Figure S12 [file cddis2016116x15.tif]
